# Eukaryotic Microbiomes of Membrane-Attached Biofilms in Membrane Bioreactors Analyzed by High-Throughput Sequencing and Microscopic Observations

**DOI:** 10.1264/jsme2.ME17112

**Published:** 2018-03-29

**Authors:** Tomohiro Inaba, Tomoyuki Hori, Yuya Sato, Tomo Aoyagi, Dai Hanajima, Atsushi Ogata, Hiroshi Habe

**Affiliations:** 1 Environmental Management Research Institute, National Institute of Advanced Industrial Science and Technology (AIST) 16–1 Onogawa, Tsukuba, Ibaraki 305–8569 Japan; 2 Dairy Research Division, Hokkaido Agricultural Research Center, National Agricultural and Food Research Organization (NARO) 1 Hitsujigaoka, Sapporo, Hokkaido 062–8555 Japan

**Keywords:** Membrane bioreactor, Eukaryotic microbiome, Biofilm, Biofouling, High-throughput sequencing

## Abstract

Limited information is currently available on the contribution of eukaryotes to the reactor performance of membrane bioreactors (MBRs). Using high-throughput Illumina sequencing of 18S rRNA genes and microscopic observations, we investigated eukaryotic microbiomes in membrane-attached biofilms in MBRs treating piggery wastewater. Protozoa preying on bacteria were frequently detected under stable conditions when membrane clogging was suppressed. However, the eukaryotes preying upon protozoa became predominant in biofilms when membrane fouling rapidly progressed. We herein demonstrated that a comprehensive investigation of eukaryotic microbiomes using high-throughput sequencing contributes to a better understanding of the microbial ecology involved in wastewater treatment.

The performance of biological wastewater treatment is closely associated with the abundance and composition of microbiomes in activated sludge. Therefore, studies on both eukaryotic and prokaryotic sludge microorganisms as performance indicators are necessary. In order to identify the diverse microbial populations or key microbial species in sludge, bacterial and archaeal microbiomes have been intensively examined using the high-throughput sequencing of 16S rRNA genes (15, 19–22). However, few attempts have been made to investigate eukaryotic microbiomes using a molecular approach. In the biological wastewater treatment process, eukaryotic microorganisms contribute to sludge sedimentation and improve water quality by predating upon prokaryotes (16), while non-predatory eukaryotes, such as *Fungi* and *Rhizaria*, have been suggested to play roles in nitrogen removal (12). Thus, the high-throughput sequencing of 18S rRNA genes will lead to a better understanding of the roles of eukaryotic microbiomes in the sludge ecosystem.

A membrane bioreactor (MBR), which combines membrane separation processes and activated sludge treatments, provides some advantages over the traditional activated sludge method, including a smaller installation area, more efficient solid-liquid separation, less excess sludge, and higher quality of treated wastewater (8, 26). In contrast, biotic membrane fouling (so-called biofouling) caused by the formation of biofilms on filtration membranes is a persistent issue in MBRs (10). In recent years, the bacterial microbiomes, chemical compositions, and three-dimensional structures of fouling-related biofilms have been investigated (5, 25, 28, 29). We previously revealed that the bacterial microbiomes of fouling-related biofilms and their developmental processes differed depending on the organic loading rates in MBRs (5). Although bacterial microbiomes and their roles in MBRs are currently being investigated, the relationship between eukaryotic microbiomes and membrane fouling remains unclear. In the present study, eukaryotic microbiomes in membrane-attached biofilms during MBR operation were investigated using high-throughput Illumina sequencing of 18S rRNA genes.

A 27-L bench-top MBR was installed to treat piggery wastewater, in which seed sludge from a municipal wastewater treatment plant (Kinu aqua-station, Ibaraki, Japan) was used as a starter. The detailed experimental set up is described in our accompanying study (6). The reactor conditions in MBR operation were divided into three conditions based on the rate of the increase in transmembrane pressure (™P) (an indicator of the progression of membrane fouling): 1) non-acclimatized unstable conditions under which rapid membrane clogging was observed (™P-increasing rate: 7.2 kPa d^−1^), 2) acclimatized stable conditions under which the ™P-increasing rate is low (0.9 kPa d^−1^), and 3) deteriorative conditions under which the highest ™P-increasing rate was observed by increasing the organic load (11.7 kPa d^−1^) (see the accompanying study [6]). The eukaryotic microbiomes that formed in biofilms on filtration membranes under each of these conditions were investigated. Regarding microscopic observations, differential interference contrast (DIC) and three-dimensional images of microbes on fouled membranes were observed with an Axio Observer Z1 (Carl Zeiss, Jena, Germany) equipped with an oil immersion objective (Plan-Apochromat 63×/1.40; Carl Zeiss) and laser scanning microscope (LSM880; Carl Zeiss) equipped with a 40×/0.80 numerical aperture Achroplan W water immersion objective (Carl Zeiss), respectively (4, 5, 14). Regarding the high-throughput sequencing analysis, total DNA samples were extracted from membrane-attached biofilms and sludge samples under each condition and used as templates for PCR amplification. The V4 region of 18S rRNA genes was amplified with universal primers (TAReuk454F and TAReuk454R) modified by the addition of an adaptor sequence and 12-base barcodes for multiplex MiSeq sequencing (11, 24). Illumina sequencing was performed on 18S rRNA gene amplicons according to a previously described method (1). Raw sequence data obtained in this study were deposited into the sequence read archive of the DNA databank of Japan (DDBJ) database (http://www.ddbj.nig.ac.jp/) under the accession number DRA005905. The removal of internal control, low-quality (Q<20), and chimeric sequences, and the assembly of paired-end reads were performed using previously described procedures based on the SILVA database (7, 17, 27). The sequences in each library were phylogenetically characterized using QIIME software with the SILVA database (3). Operational taxonomic units (OTUs) were defined using a sequence identity cut-off of 97%. Alpha-diversity indices (*i.e.*, Chao1, Shannon, and Simpson reciprocal) and weighted UniFrac distances for a principal coordinate analysis (PCoA) were calculated based on an equal number (*n*=9,838) of sequences using QIIME software (Table S1 and Fig. S1). The results of the alpha-diversity indices and PCoA plot analysis showed that membrane-attached biofilms and sludge both formed different eukaryotic microbiomes in response to the reactor conditions. In order to gain further insights into the eukaryotes associated with reactor conditions, the closest relative of each OTU was identified through an NCBI BLAST search (http://blast.ncbi.nlm.nih.gov/), and the ten most abundant OTUs in membrane-attached biofilms are shown in Table 1.

Under non-acclimatized unstable conditions, *Fungi* accounted for more than 59.6% of the eukaryotic microbiome, and the most abundant organism OTU 117 (*Hyaloraphidium curvatum* [accession no. NG017172; 88% sequence similarity]) belonged to the phylum *Chytridiomycota*. Microorganisms resembling chytrids in form were detected by microscopic observations of membrane-attached biofilms that formed under unstable conditions (Fig. S2). A previous study suggested that dimorphic fungi, which belong to the phyla *Mucoromycota* and *Ascomycota*, are potentially involved in biofouling (13). Most of the ten most abundant OTUs exhibited low similarities (less than ~85% [11])] to sequences in the database, suggesting that unknown eukaryotes, particularly *Fungi*, contribute to a rapid increase in ™P, *i.e.* rapid membrane clogging, under unstable conditions. Moreover, the composition of the ten most abundant OTUs, except OTU 117, differed between the biofilm and sludge (Tables 1 and S2), indicating the specific distributions of these dominant eukaryotes in biofilms.

Unknown *Fungi* were not frequently detected under acclimatized stable conditions; eukaryotes belonging to the superphylum *Alveolata* (including the phyla *Ciliphora*, *Apicomplexa*, and *Dinoflagellata*) and unranked taxon *Opisthokont* accounted for 75.1% of the biofilm eukaryotic microbiome (Table 1). The most abundant taxon, OTU 17845 (*Campanella umbellaria* [AF401524; 88% similarity]), was a protozoan classified into the phylum *Ciliophora*. Protozoan-like microorganisms resembling ciliates in form were frequently observed in the membrane-attached biofilms that formed under stable conditions (Fig. 1A). Ciliates are phagotrophic protists that prey upon bacteria and yeast (9, 23); therefore, predation by ciliates in biofilms may have contributed to a decrease in the ™Pincreasing rate, *i.e.*, they potentially inhibited membrane fouling caused by thick biofilm formation. Moreover, metazoanlike microorganisms that were rarely detected under unstable conditions were present. OTUs 4403 (*Habrotrocha bidens* [KM043258; 100% similarity]) and 18381 (*Lecane bulla* [DQ297698; 98% similarity]) were bdelloid rotifers belonging to the phylum *Rotifera*, and some rotifers are predatory metazoans (2). *Lecane bulla*-like microorganisms were found in biofilms and visualized by confocal reflection microscopy (Fig. 1B). These results strongly suggested that a large number of predatory eukaryotes were localized on filtration membranes and that their predatory activities toward bacteria may suppress the formation of membrane-attached biofilms. Although the composition of abundant OTUs in biofilms was similar to that in sludge (Fig. S1 and Table S2), some OTUs presumed to be predatory eukaryotes (*e.g.*, OTU 17845 and OTU 18381) were abundant in biofilms (Table 1), suggesting that predation by eukaryotes likely occurred in biofilms under stable conditions.

Under deteriorative conditions induced by overloading, the most abundant OTU 1587 (*Tokophrya infusionum* [JQ723984; 94% similarity]) accounted for 73.49% of the biofilm eukaryotic microbiome, and its relative abundance was 620-fold higher on membranes than in sludge (Table 1). *Tokophrya infusionum* is a sessile suctorian and a known predator of living ciliates (18). The relative abundance of OTU 17845, which was presumed to be a ciliate-like eukaryote, was markedly lower under deteriorative conditions than under stable conditions. Moreover, the most abundant OTUs were not specific to biofilms; they were also abundant in sludge (Table S2). These results suggest that an increase in eukaryote predation upon protozoa (OTU 1587) markedly decreased the abundance of protozoa preying on bacteria, and this may be related to the rapid increase observed in the ™P-increasing rate.

The high-throughput Illumina sequencing of 18S rRNA genes revealed that eukaryotic microbiomes in membrane-attached biofilms are related to reactor conditions, and may have played a role in membrane fouling. ™P increased rapidly when *Fungi* and eukaryotes preying upon protozoa dominated the biofilm microbiome. In contrast, ™P increases were suppressed when protozoa preying upon bacteria were dominant in the biofilm microbiome. The results of the present study suggest that eukaryotic microbiomes in biofilms are an important factor for the development of membrane fouling during MBR operation. Combined with the findings of our recent study of a distinct relationship between the bacterial microbiome and reactor conditions (see the accompanying study [6]), the present results indicate that comprehensive investigations covering eukaryotes via the high-throughput Illumina sequencing of 18S rRNA genes are essential for gaining a deeper understanding of the predator-prey interactions that are closely associated with the reactor performance of MBRs. Further studies on the relationships among eukaryotic microbiomes, reactor conditions, and membrane fouling are needed in order to elucidate the ecological roles of eukaryotic microbiomes in MBRs.

## Supplementary Material





## Figures and Tables

**Fig. 1:**
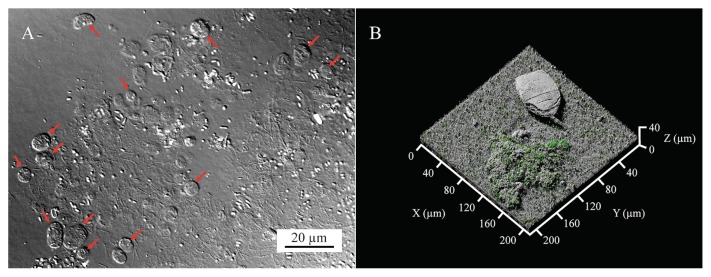
Microscopic images of eukaryotes in a membrane-attached biofilm. (A) A DIC microscopic image of microbes on a fouled membrane. Samples for DIC microscopy were prepared by scraping the 26-kPa membrane-attached biofilm formed under stable conditions. Red arrows indicate organisms presumed to be ciliates based on morphology. The bar shows 20 μm. At least three images were taken, and a representative image is shown. (B) Three-dimensional image of the 6-kPa membrane-attached biofilm, visualized by continuous-optimizing confocal reflection microscopy (COCRM). The membrane sample taken under stable conditions was washed once with water and installed at the bottom of an observation chamber filled with water. Microscopic observations using COCRM were performed with LSM880. Gray indicates a reflected-light image of the physical body, and green and red indicate live and dead microbial cells, respectively. Live/dead staining was performed with SYTO9/propidium iodide dyes. Imaging was repeated six times at random positions on the biofilm, and a representative image including a eukaryote is shown.

**Table 1:** Ten most abundant operational taxonomic units (OTUs) in biofilms under unstable, stable, and deteriorative conditions.

OTU ID	Eukaryotic microorganisms		Accession No.	Identity (%)	Relative abundance^a^ (%)	Fold change^b^

	Kingdom/phylum	Species				
**Unstable (non-acclimatized)**						
117	*Fungi*	*Hyaloraphidium curvatum*	NG017172	84	25.51	0.5
12482	*Fungi*	*Mortierella wolfii*	KR819140	84	23.24	48.6
12487	*Ciliphora*	*Platyophrya vorax*	AF060454	99	5.83	—
6662	*Fungi*	*Keissleriella quadriseptata*	AB797303	89	5.05	61.0
10604	*Miozoa*	*Blastodinium mangini*	JX473656	86	3.57	—
18203	*Cercozoa*	*Cercomonas agilis*	AY748806	85	3.48	—
11607	*Ochrophyta*	*Poterioochromonas malhamensis*	KY432752	95	3.19	—
10603	*Fungi*	*Neocallimastix frontalis*	HQ585897	84	2.50	6.9
5249	*Fungi*	*Paramicrosporidium vannellae*	JQ796368	85	1.68	55.1
2717	*Fungi*	*Cyllamyces aberensis*	DQ536481	84	1.65	163.6
**Stable (acclimatized)**						
17845	*Ciliphora*	*Campanella umbellaria*	AF401524	88	51.06	2.5
14179	Opisthokont*	*Salpingoeca tuba*	HQ026774	87	16.92	2.6
4403	*Metazoa*	*Habrotrocha bidens*	KM043258	100	5.04	0.1
7520	Opisthokont*	*Anurofeca richardsi*	AF070445	94	4.15	6.0
18381	*Metazoa*	*Lecane bulla*	DQ297698	98	1.76	7.1
467	*Fungi*	*Paramicrosporidium vannellae*	JQ796368	86	1.72	0.8
117	*Fungi*	*Hyaloraphidium curvatum*	NG017172	84	1.49	0.5
19310	*Dinoflagellata*	*Exuviaella pusilla*	DQ388459	91	1.08	4.9
6169	Opisthokont*	*Sphaeroeca leprechaunica*	KJ631042	98	1.02	503.6
7941	*Apicomplexa*	*Ascogregarina armigerei*	DQ462459	82	0.85	2.3
**Deteriorative (acclimatized, but overloading)**						
1587	*Ciliphora*	*Tokophrya infusionum*	JQ723984	94	73.49	620.4
17845	*Ciliphora*	*Campanella umbellaria*	AF401524	88	8.09	0.2
16518	*Metazoa*	*Macrobiotus sapiens*	DQ839601	96	3.65	0.7
4403	*Metazoa*	*Habrotrocha bidens*	KM043258	100	2.23	0.2
16474	*Fungi*	*Hyaloraphidium curvatum*	NG017172	84	1.72	0.3
2632	*Fungi*	*Candida oregonensis*	AB013545	90	1.13	1.1
18381	*Metazoa*	*Lecane bulla*	DQ297698	98	0.70	3.5
19310	*Dinoflagellata*	*Exuviaella pusilla*	DQ388459	91	0.67	0.2
4361	*Ciliphora*	*Tokophrya infusionum*	JQ723984	90	0.61	458.5
3603	*Cercozoa*	*Orciraptor agilis*	KF207875	81	0.58	0.2

a Relative abundance showed an average based on two independent determinations.

b Fold changes relative to the planktonic state (sludge) are shown.

* Phylum level classification is uncertain, and classified as the unranked taxon *Opisthokont*
