# Supplementary material for: Eukaryotic Microbiomes of Membrane-Attached Biofilms in Membrane Bioreactors Analyzed by High-Throughput Sequencing and Microscopic Observations
Source: Microbes Environ. 2018 Mar 29;33(1):98–101. doi: 10.1264/jsme2.ME17112 (PMC5877349; doi:10.1264/jsme2.ME17112)
Supplement: Supplementary file 1 [file 33_98_s1.pdf]

Supplemental information

Supplemental Table S1. Summary of Illumina sequencing results.

Sample		Loading conditions		Number of sequences	Number of OTUs	C_X[a]	Diversity indices[b]		
		Volumetric load (mgCOD $L^{-1}d^{-1}$)	HRT (d)				Chao1	Shannon	1/Simpson
Piggery wastewater				41262±418	96±15	0.998±0.0004	208±28	1.77±0.12	1.76±0.10
Seed				33235±168	473±0	0.986±0.0001	919±0	4.54±0.29	7.61±2.20
Biofilm	Unstable	704	6	30581±1922	350±20	0.988±0.0014	1046±113	4.56±0.05	7.93±0.10
	Stable	704	6	20656±418	269±81	0.986±0.0027	998±177	3.29±0.37	3.58±0.99
	Deteriorative	1,407	3	50017±418	264±8	0.995±0.0005	494±83	2.03±0.12	1.84±0.11
Sludge	Unstable	704	6	23661±1148	437±33	0.991±0.0009	680±29	2.83±0.03	2.96±0.01
	Stable	704	6	34694±2732	955±35	0.980±0.0001	1375±22	3.78±0.12	4.44±0.16
	Deteriorative	1,407	3	30581±1922	933±43	0.985±0.0014	1173±93	3.69±0.21	4.01±0.33

[a]Calculated from the equation $C_X = 1 - (n/N)$, where “n” is the number of singleton OTUs and N is the total number of sequences.
[b]Each index was calculated based on an equal number of sequences (*n*=9,838). All data presented are based on the average of two replicates.

Supplemental Table S2. Ten most abundant operational taxonomic units (OTUs) in sludge under unstable, stable, and deteriorative conditions.

OTU ID	Eukaryotic microorganisms		Accession No.	Identity (%)	Relative abundance[a] (%)	Fold change[b]
	Kingdom/ phylum	Species				
**Unstable (non-acclimatized)**						
117	*Fungi*	*Hyaloraphidium curvatum*	NG017172	84	53.14	2.1
6492	*Metazoa*	*Isohypsibius granulifer*	EF620403	98	25.38	71.3
11958	*Cercozoa*	*Rhogostoma minus*	LC032468	97	1.92	6.6
17603	*Metazoa*	*Polyarthra remata*	DQ297716	95	1.33	2.6
14625	*Fungi*	*Hyaloraphidium curvatum*	NG017172	82	1.23	2.4
4676	*Metazoa*	*Isohypsibius granulifer*	EF620403	95	1.13	90.2
16984	*Fungi*	*Hyaloraphidium curvatum*	NG017172	83	1.13	2.5
10633	*Metazoa*	*Isohypsibius granulifer*	EF620403	95	1.12	—
13663	*Fungi*	*Hyaloraphidium curvatum*	NG017172	83	0.98	5.2
12713	*Cercozoa*	*Cryothecomonas aestivalis*	AF290541	82	0.93	24.3
**Stable (acclimatized)**						
4403	*Metazoa*	*Habrotrocha bidens*	KM043258	100	43.22	8.6
17845	*Ciliphora*	*Campanella umbellaria*	AF401524	88	20.61	0.4
14179	*Opisthokont**	*Salpingoeca tuba*	HQ026774	87	6.47	0.4
117	*Fungi*	*Hyaloraphidium curvatum*	NG017172	84	3.19	2.1
16432	*Metazoa*	*Habrotrocha bidens*	KM043258	100	2.79	8.3
1610	*Fungi*	*Mortierella clonocystis*	HQ667479	85	2.20	2.7
467	*Fungi*	*Paramicrosporidium vannellae*	JQ796368	86	2.08	1.2
3603	*Cercozoa*	*Orciraptor agilis*	KF207875	81	1.85	4.4
18072	*Metazoa*	*Encentrum astridae*	DQ297695	98	1.66	5.8
19347	*Ciliphora*	*Opercularia microdiscum*	AF401525	88	1.66	2.9
**Deteriorative (acclimatized but overloading)**						
17845	*Ciliphora*	*Campanella umbellaria*	AF401524	88	48.04	5.9
4403	*Metazoa*	*Habrotrocha bidens*	KM043258	100	14.80	6.6
16518	*Metazoa*	*Macrobiotus sapiens*	DQ839601	96	5.47	1.5
16474	*Fungi*	*Hyaloraphidium curvatum*	NG017172	84	5.03	2.9
19310	*Dinoflagellata*	*Exuviaella pusilla*	DQ388459	91	3.26	4.9
3603	*Cercozoa*	*Orciraptor agilis*	KF207875	81	2.51	4.3
117	*Fungi*	*Hyaloraphidium curvatum*	NG017172	84	2.02	7.7
467	*Fungi*	*Paramicrosporidium vannellae*	JQ796368	86	1.97	4.7
19347	*Cercozoa*	*Opercularia microdiscum*	AF401525	88	1.52	10.3
16432	*Metazoa*	*Habrotrocha bidens*	KM043258	100	1.06	8.5

[a]Relative abundance represents average values of two independent determinations.
[b]Fold changes relative to the biofilm are shown.
*Phylum level classification is uncertain, and classified as the unranked taxon *Opisthokont*.

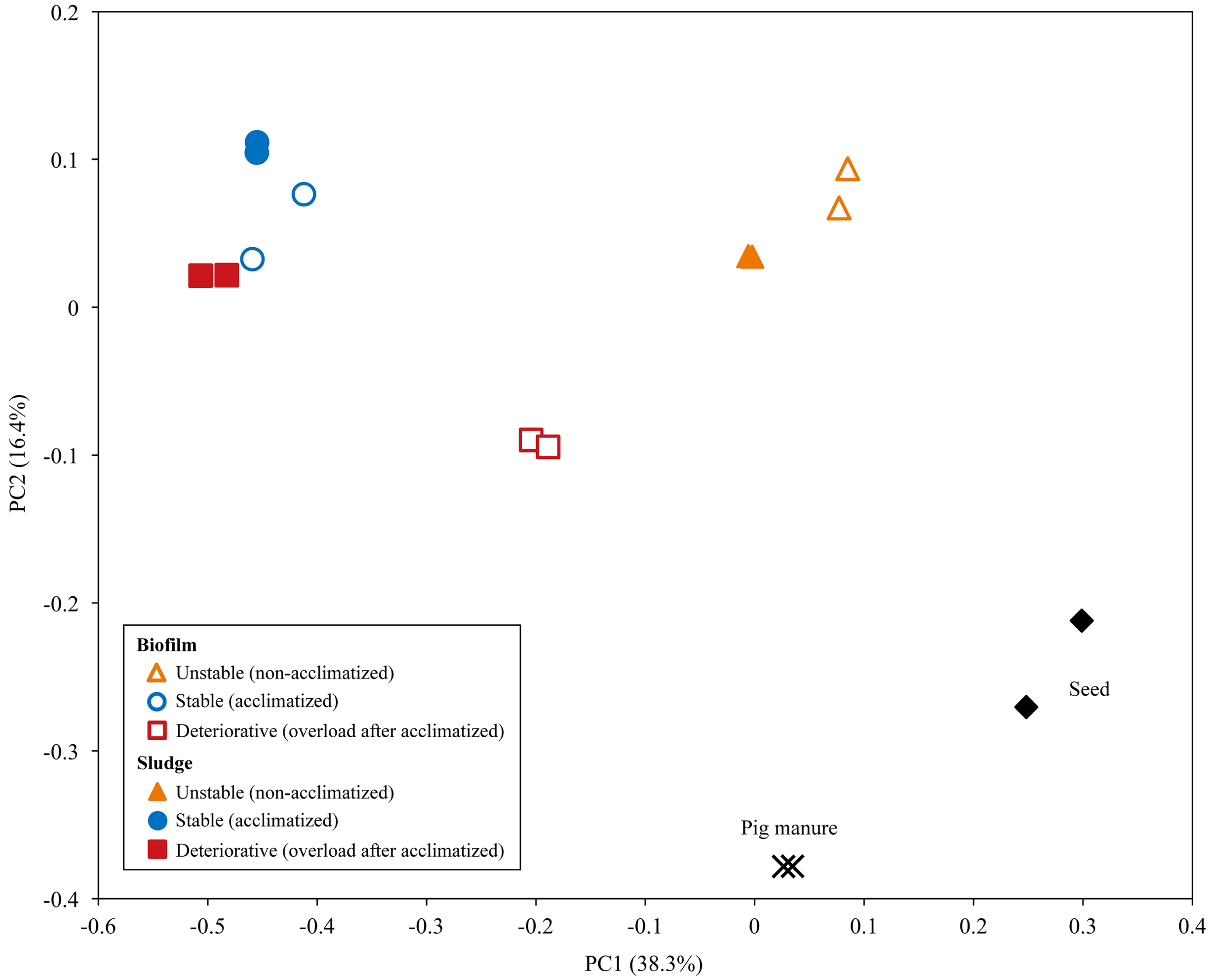

Supplemental Fig. S1. Comparison of biofilm and sludge microbiomes during piggery wastewater treatment. A principal coordinate analysis (PCoA) scatter plot of 18S rRNA genes obtained via Illumina sequencing. Weighted UniFrac distances were calculated based on an equal number (n=9,838) of sequences. Open and closed symbols indicate sludge and biofilm microbiomes, respectively, while the symbol type indicates reactor conditions.
